# Gln40 deamidation blocks structural reconfiguration and activation of SCF ubiquitin ligase complex by Nedd8

**DOI:** 10.1038/ncomms10053

**Published:** 2015-12-03

**Authors:** Clinton Yu, Haibin Mao, Eric J. Novitsky, Xiaobo Tang, Scott D. Rychnovsky, Ning Zheng, Lan Huang

**Affiliations:** 1 Department of Physiology and Biophysics, University of California, Irvine, California 92697, USA; 2 Department of Pharmacology and Howard Hughes Medical Institute, University of Washington, Seattle, Washington 98195, USA; 3 Department of Chemistry, University of California, Irvine, California 92697, USA

## Abstract

The full enzymatic activity of the cullin-RING ubiquitin ligases (CRLs) requires a ubiquitin-like protein (that is, Nedd8) modification. By deamidating Gln40 of Nedd8 to glutamate (Q40E), the bacterial cycle-inhibiting factor (Cif) family is able to inhibit CRL E3 activities, thereby interfering with cellular functions. Despite extensive structural studies on CRLs, the molecular mechanism by which Nedd8 Gln40 deamidation affects CRL functions remains unclear. We apply a new quantitative cross-linking mass spectrometry approach to characterize three different types of full-length human Cul1–Rbx1 complexes and uncover major Nedd8-induced structural rearrangements of the CRL1 catalytic core. More importantly, we find that those changes are not induced by Nedd8(Q40E) conjugation, indicating that the subtle change of a single Nedd8 amino acid is sufficient to revert the structure of the CRL catalytic core back to its unmodified form. Our results provide new insights into how neddylation regulates the conformation and activity of CRLs.

## Abstract (web summary)

Cullin-RING ubiquitin ligases (CRLs) require neddylation of their cullin scaffolds for full activity. Here the authors use a quantitative cross-linking mass spectrometry approach to characterize three different full-length human Cul1-Rbx1 complexes to shed light on how neddylation regulates the activity of CRLs.

## Graphical abstract

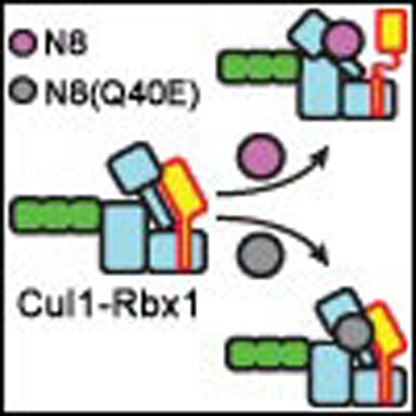

Cullin-RING ubiquitin ligases (CRLs) represent a superfamily of multi-subunit E3 ubiquitin ligases comprised of a cullin-RING catalytic core and adaptor proteins that mediate the recruitment of protein substrates^1^,^2^,^3^,^4^,^5^,^6^. Eight cullin family proteins (Cul1, Cul2, Cul3, Cul4A/B, Cul5, Cul7, Cul9 and APC2) are found in humans, each functioning as a scaffold on which a variety of CRLs are assembled. The SCF/CRL1 (Skp1–Cul1–F-box protein) complex represents the prototypical CRL E3, which uses Cul1–Rbx1 as the catalytic core^2^,^7^,^8^. The Cul1 scaffold binds the Skp1 adaptor and the Rbx1 RING subunit at its N-terminal and C-terminal domains, respectively. Skp1 in turn docks F-box proteins, which are substrate receptors that confer substrate specificity to the SCF, while the RING-finger domain of Rbx1 engages ubiquitin-charged E2, mediating the transfer of ubiquitin to the F-box protein-bound substrate. A reconstructed structure model of the SCF based on crystal structures of several overlapping sub-complexes reveals an elongated E3 platform, in which the F-box protein is separated from the Rbx1-bound E2 by a ∼50-Å distance^9^.

Covalent conjugation of ubiquitin-like protein Nedd8 (that is, neddylation) to a specific Lysine (Lys720) of Cul1 has been shown to promote both E2 recruitment and subsequent ubiquitin transfer, thereby stimulating the E3 activity of SCF ligases^2^,^10^,^11^,^12^,^13^. Although the intact neddylated Cul1–Rbx1 complex remains recalcitrant to crystallization, crystal structures of a truncated C-terminal domain of Cul5 in complex with Rbx1 have shed light on the effects of neddylation on the conformation of the cullin-RING catalytic core^14^. In the unneddylated form, the Cul5^CTD^–Rbx1 complex adopts a 'closed' conformation in which the RING-finger domain of Rbx1 is nestled within a hydrophobic pocket of Cul5^CTD^. Upon neddylation, the RING-finger domain of Rbx1 is released from the pocket, deemed the 'open' state, but remains tethered by its N-terminus to Cul5, presumably allowing the extended RING-finger to sample the three-dimensional (3D) space around Cul5. This conferred flexibility has been proposed to enable Rbx1 to close the distance between substrate and E2, facilitating the transfer of ubiquitin from E2 to substrate protein.

Notably, the cycle-inhibiting factors (Cifs) found in many pathogenic Gram-negative bacteria can irreversibly deamidate a specific glutamine residue (Gln40) of Nedd8 and convert it to glutamate^15^. This Q40E modification has no effect on cullin neddylation, but can effectively abolish the E3 activity of CRLs and affect proper cullin deneddylation by the COP9 signalosome^15^,^16^,^17^,^18^. These observations raise an intriguing question as to how the subtle change of a single Nedd8 amino acid is able to negate the effect of neddylation in remodelling the ∼100-kDa CRL catalytic core. In the structure of the neddylated Cul5^CTD^–Rbx1 complex, Gln40 of Nedd8 is close to the isopeptide bond between Nedd8 and Cul5 and partially sandwiched between the two proteins. The amide group in the Gln40 side chain, however, is exposed to the solvent and does not participate in any hydrogen bond interactions^14^. The molecular mechanism by which Nedd8 Gln40 deamidation alters CRL functions remains elusive.

Recently, cross-linking mass spectrometry (XL-MS) has risen as a powerful method to study protein–protein interactions and characterize the structure of large protein complexes^19^,^20^,^21^,^22^,^23^,^24^,^25^,^26^,^27^,^28^. In comparison with X-ray crystallography or NMR, XL-MS approaches have much less restriction on sample preparation due to its sensitivity, flexibility and versatility, and are capable of capturing the dynamic states of large, heterogeneous protein structures. By stabilizing transient interactions, chemical cross-linking preserves various structural states of dynamic complexes, yielding a representation that describes the average state of a protein complex and providing a complementary set of structural data different from that obtained from rigid state data analyses such as X-ray crystallography. Recently, we have developed a new class of cross-linkers, that is, sulfoxide-containing MS-cleavable cross-linking reagents, to enable simplified and unambiguous identification of cross-linked peptides using multistage tandem mass spectrometry (MS^*n*^)^29^,^30^,^31^. These new types of cross-linkers are robust and reliable, and have been successfully applied to define protein–protein interactions both *in vitro*^22^,^29^,^30^ and *in vivo*^30^. To establish a robust quantitative XL-MS (QXL-MS) platform to study dynamic protein complexes, we have then developed a pair of stable isotope-labelled amine reactive cross-linkers (that is, d_0_- and d_10_-labelled dimethyl-disuccinimidyl sulfoxide (DMDSSO)), which allow simultaneous identification and quantitation of cross-linked peptides^31^. In combination with quantitative analysis, XL-MS can determine dynamic conversion between the average states of protein complexes under different conditions.

Here we employ this DMDSSO-based QXL-MS strategy to define the structural changes of full-length Cul1–Rbx1 modified by either wild-type Nedd8 or its Q40E mutant, which is the product of Gln40 deamidation. Quantitative similarities and differences in cross-linked peptide abundances can be attributed to the changes in protein complex structures under different conditions, as the occurrences of spatially proximal amino-acid residues suited for cross-linking is directly dependent on the 3D structural conformation of these complexes. Our results have provided new insights on how Nedd8 modification impacts the topology of Cul1–Rbx1 and the effect of Nedd8 Gln40 deamidation on the structure of the activated CRL core.

## Results

### Reconstitution of SCF E3 activity with intact proteins

To enhance the solubility and stability of Cul1, we removed two short segments (see details in Methods) of Cul1 which were not visible in the crystal structure of Cul1–Rbx1 complex (PDB: 1LDJ)^32^, drastically improving protein behaviour. This truncated Cul1 and Rbx1^16–108^ were co-expressed and purified from *Escherichia coli*. The purified Cul1–Rbx1 was conjugated to wild-type Nedd8 to yield neddylated Cul1–Rbx1 complex (Nedd8∼Cul1–Rbx1). The Q40E mutant Nedd8, in which Gln40 was replaced with Glu40 to mimic deamidated Nedd8, can also be efficiently conjugated to Cul1–Rbx1 to form Nedd8(Q40E)∼Cul1–Rbx1. Both neddylated Cul1–Rbx1 samples were purified with affinity-tagged Nedd8 after neddylation reaction to remove the unmodified species.

**Figure 1:**
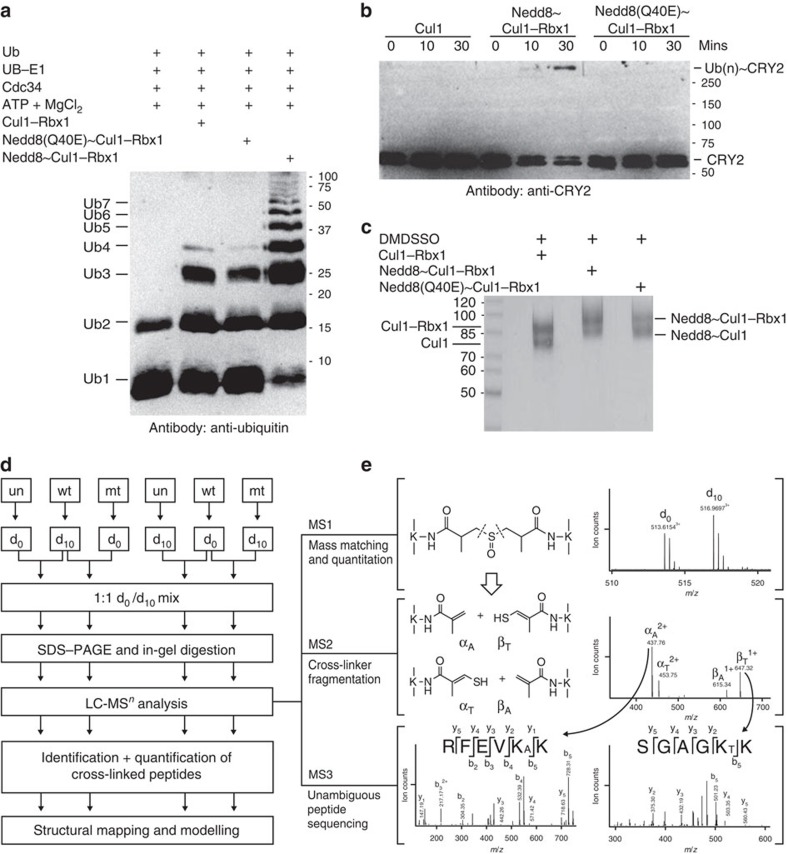
Biochemical assays for ubiquitin ligase activity and general quantitative XL-MS experimental workflow. (**a**) Comparisons of ubiquitin ligase activities of different Cul1–Rbx1 variants on free ubiquitin chain assembly. Synthesized unanchored polyubiquitin chains were detected by anti-ubiquitin western blot. Highly efficient ubiquitin synthesis was only detected in the presence of Nedd8–Cul1–Rbx1 (lane 4). For visual clarity, ubiquitin polymers are simply abbreviated as Ub(n) (for example, Ub2 as ubiquitin dimer, Ub3 as ubiquitin trimer and so on). (**b**) Comparisons of ubiquitin ligase activities of different Cul1–Rbx1 variants on CRY2 ubiquitination. Ubiquitination reactions were quenched at indicated time points. Ubiquitinated CRY2 was detected using an anti-CRY2 antibody. Successful ubiquitination of CRY2 occurs only in the presence of Nedd8–Cul1–Rbx1 (lane 6). (**c**) SDS–PAGE analysis of DMDSSO cross-linked Cul1–Rbx1, Nedd8∼Cul1–Rbx1 and Nedd8(Q40E)∼Cul1–Rbx1 complexes. (**d**) d_0_/d_10_-DMDSSO-based quantitative XL-MS workflow for identifying and quantifying cross-linked peptides of Cul1–Rbx1 complexes. The three types of Cul1–Rbx1 complexes, that is, un, unneddylated; wt, wild-type neddylated; mt, mutant Q40E-neddylated, were first cross-linked by DMDSSO separately, two of which were then selected for mixing before SDS–PAGE. Four types of mixing were made to obtain sufficient pairwise comparison among the three samples. Gel bands representing cross-linked protein complexes were subsequently excised and in-gel digested before LC-MS^*n*^ analysis for identification and quantification. (**e**) Representative MS^*n*^ analysis of DMDSSO interlinked peptides. MS^1^ spectrum shows the detection of a pair of d_0_-DMDSSO and d_10_-DMDSSO cross-linked peptides (*m*/*z* 513.6154^3+^ and *m*/*z* 516.9697^3+^), whose spectral relative abundance ratio is used for quantitation. MS^2^ analysis of the d_0_-DMDSSO interlinked peptides α–β (*m*/*z* 513.6154^3+^) yielded two peptide fragment pairs: α_A_/β_T_ (*m*/*z* 437.76^2+^/647.32^1+^) and α_T_/β_A_ (*m*/*z* 453.75^2+^/615.34^1+^), confirming its cross-link type as an interlink. Subsequent MS^3^ analyses of the α_A_ (*m*/*z* 437.76^2+^) and β_T_ (647.32^1+^) ions produced series of y and b ions that enabled unambiguous identification of α_A_ as RFEVK_A_K of Rbx1 and β_T_ as SGAGK_T_K of Rbx1. Integration of the MS^*n*^ (that is, MS^1^, MS^2^ and MS^3^) data has confirmed the d_0_-DMDSSO cross-linked peptide as an intrasubunit interlink between K19 and K25 of Rbx1. K_A_, alkene modified lysine; K_T_, unsaturated thiol modified lysine.

To understand ubiquitin ligase activities of different forms of Cul1–Rbx1, we have employed two *in vitro* ubiquitination assays: free ubiquitin chain assembly and ubiquitination of cryptochrome 2 (CRY2). CRY2, a key regulator of circadian rhythm, is a well-characterized substrate of the SCF^FBXL3^ ubiquitin ligase^33^,^34^,^35^,^36^. In both assays, we used Cdc34, the canonical E2 of Cul1. Consistent with previous reports, Cul1–Rbx1 can promote substrate-independent free ubiquitin chain assembly, while Nedd8∼Cul1–Rbx1 significantly enhanced the reaction kinetics (Fig. 1a)^37^. In contrast, Nedd8(Q40E)∼Cul1–Rbx1 only exhibited comparable activity to unneddylated Cul1–Rbx1, which was much weaker than that of Nedd8∼Cul1–Rbx1. This is consistent with the discovery that deamidation of Q40 in Nedd8 abolishes the ligase activity of neddylated Cul1–Rbx1 (refs 15, 16).

We further confirmed this observation with an *in vitro* ubiquitination assay of CRY2. As shown in Fig. 1b, polyubiquitin chains were formed on CRY2 in the presence of Nedd8∼Cul1–Rbx1. In contrast, neither unneddylated nor Nedd8(Q40E)-modified Cul1–Rbx1 complexes were able to catalyse ubiquitination of CRY2. This further confirms that Nedd8∼Cul1–Rbx1 is the only active form and that deamidation of Q40 in Nedd8 can in fact abrogate its activity. Compared with the free ubiquitin chain assembly assay, the polyubiquitin chain synthesis on CRY2 was highly processive, as the ubiquitinated CRY2 band was observed at the top of the gel as shown by western blot analysis. Taken together, our results have demonstrated neddylation is essential for the activation of the Cul1–Rbx1 complex in protein ubiquitination. Importantly, distinct functional disparity between Nedd8∼Cul1–Rbx1 and Nedd8(Q40E)∼Cul1–Rbx1 have been further validated, and only the former is the active E3 ligase for protein ubiquitination.

### QXL-MS strategy

To understand molecular details underlying the functional differences between different forms of Cul1–Rbx1 complexes, we have employed a QXL-MS strategy based on a newly developed pair of stable isotope-coded MS-cleavable cross-linkers, d_0_-DMDSSO and d_10_-DMDSSO^31^ (Supplementary Fig. 1a,b), to examine the structural similarities and dissimilarities between these complexes. Concurrent usage of these two cross-linking reagents enables quantitative comparisons between the 3D structures of protein complexes under various conditions. To establish the QXL-MS workflow for comprehensive structural comparisons among unneddylated (un), wild-type neddylated (wt) and Q40E mutant neddylated (mt) Cul1–Rbx1 complexes, cross-linking conditions were first optimized through *in vitro* cross-linking of the three protein complexes with various concentrations of either d_0_-DMDSSO or d_10_-DMDSSO for different amounts of reaction times. Cross-linking efficiency was then evaluated by separating the resulting cross-linked products using one-dimensional SDS–polyacrylamide gel electrophoresis (SDS–PAGE). As shown in Fig. 1c, the resulting cross-linked products correspond well to respective molecular weights of these three complexes with ∼50% cross-linking efficiency. In addition, we have determined that d_0_- and d_10_-DMDSSO reacted with Cul1–Rbx1 complexes with similar efficiency as illustrated in Supplementary Fig. 1c, also reflected in previous testing on standard proteins^31^. These results demonstrate that d_0_- and d_10_-labelled DMDSSO are well suited for QXL-MS analysis of these protein complexes.

To enable sufficient comparisons among the three different types of protein complexes with the minimal number of samples for analysis, we have strategically selected Nedd8∼Cul1–Rbx1 as the cross-sample reference in the pairwise comparison experiments. As illustrated in Fig. 1d, d_10_-DMDSSO cross-linked Nedd8∼Cul1–Rbx1 was mixed with d_0_-DMDSSO cross-linked Cul1–Rbx1 or d_0_-DMDSSO cross-linked Nedd8(Q40E)∼Cul1–Rbx1, followed by SDS–PAGE separation. The regions corresponding to expected cross-linked complexes were in-gel digested and the resulting peptides were subjected to liquid chromatography (LC)-MS^*n*^ analysis. DMDSSO cross-linked peptides were identified unambiguously based on MS^*n*^ data, that is, MS^1^, MS^2^ and MS^3^, as previously described^29^,^30^,^31^. Representative MS^*n*^ analyses of d_0_-DMDSSO and d_10_-DMDSSO interlinked peptides α–β (*m*/*z* 513.6154^3+^ and *m*/*z* 516.9697^3+^, respectively) are shown (Fig. 1e; Supplementary Fig. 2). As shown in Fig. 1e, MS^2^ analysis of the d_0_-DMDSSO interlinked peptide α–β yielded two expected fragment pairs α_A_/β_T_ (*m*/*z* 437.76^2+^/647.32^1+^) and α_T_/β_A_ (*m*/*z* 453.75^2+^/615.34^1+^), which are characteristic of DMDSSO interlinked peptides^31^, confirming the type of cross-link observed here. Subsequent MS^3^ analysis of the α_A_ fragment (*m*/*z* 437.76^2+^) produced a series of y and b ions that enabled its unambiguous identification as ^21^RFEVK_A_K^26^ of Rbx1 with K25 modified with DMDSSO alkene remnant. Similarly, MS^3^ analysis of the β_T_ fragment (*m*/*z* 647.32^1+^) identified its sequence unambiguously as ^15^SGAGK_T_K^20^ of Rbx1 with K19 modified with the unsaturated thiol remnant. Together with MS^1^ mass matching, we confidently determined this d_0_-DMDSSO cross-linked peptide as an intraprotein interlink between K19 and K25 of Rbx1. Similar MS^*n*^ analysis of the same peptide cross-linked with d_10_-DMDSSO (*m*/*z* 516.9697^3+^) further confirms and identifies the intrasubunit K–K linkage within Rbx1 (Supplementary Fig. 2). On the basis of the identical fragmentation patterns of d_0_- and d_10_-DMDSSO cross-linked peptides, our results demonstrate that d_0_- and d_10_-DMDSSO contain the same functionality and characteristics required for the unambiguous identification of their respective cross-linked peptides by MS^*n*^ analysis. Therefore, identification of either of the d_0_- or d_10_- cross-linked peptide pairs in each pairwise experiment would allow us to quantify differences in their relative abundances.

To quantify the identified d_0_- and d_10_-DMDSSO cross-linked peptides, we then determined the relative abundance ratios of corresponding peptide pairs based on their MS^1^ spectral intensities. The same pairwise comparison experiments were repeated using reversed cross-linker treatments (that is, d_0_-DMDSSO cross-linked Nedd8∼Cul1–Rbx1 was mixed with d_10_-DMDSSO cross-linked Cul1–Rbx1 or d_10_-DMDSSO cross-linked Nedd8(Q40E)∼Cul1–Rbx1) to rule out cross-linking bias due to reagent deuteration (Fig. 1d).

### Mapping XL-MS data to Cul1–Rbx1 complexes

**Figure 2:**
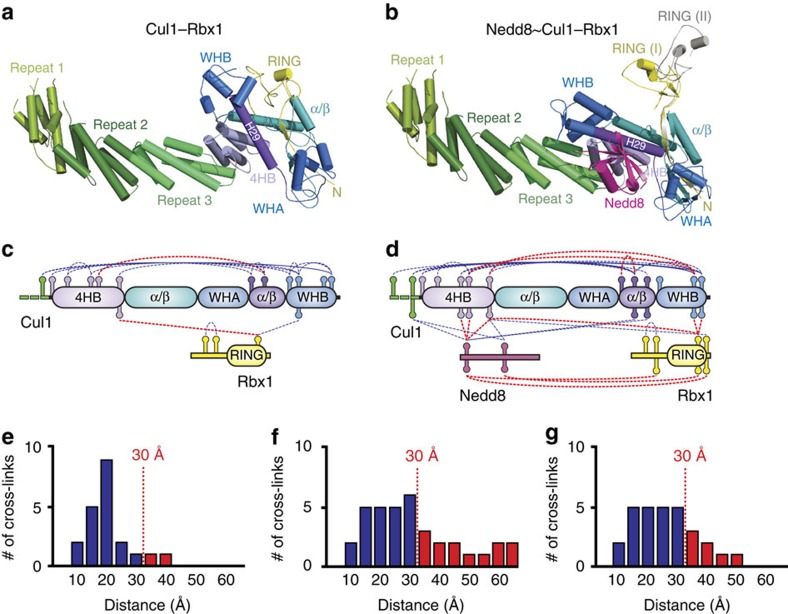
Mapping cross-link data onto current structural models of Cul1–Rbx1 complexes. (**a**) The known structure of unneddylated Cul1–Rbx1 complex. (**b**) The overlay of the two homology models of neddylated Cul1–Rbx1 derived from Nedd8∼Cul5^CTD^–Rbx1 structure, depicting two conformations of the Rbx1 RING domain with I in yellow and II in grey. On the basis of the identified interlinked peptides, the cross-link maps were generated for (**c**) Cul1–Rbx1 and (**d**) Nedd8∼Cul1–Rbx1 complexes. Note: linkages between residues with spatial distances below 30 Å are shown in blue-dotted lines, while those above 30 Å in red-dotted lines, correlating with colour-coded bar graphs in **e**–**g**. (**e**) The distribution plot of identified linkages versus their spatial distances between interlinked lysines in Cul1–Rbx1 structure. (**f**) The distribution plot of identified linkages versus their spatial distances between interlinked lysines in Nedd8∼Cul1–Rbx1 structure models. (**g**) The distribution plot of identified linkages involving only Cul1 and Nedd8 versus their spatial distances between interlinked lysines in Nedd8∼Cul1–Rbx1 structure models.

The current structural model of unneddylated Cul1–Rbx1 is described in Fig. 2a, based on a previously reported crystal structure of full-length human Cul1–Rbx1 (PDB 1LDJ)^32^. In this structure, the N-terminal domain of Cul1 consists of three helical repeats (repeat 1, 2 and 3), each comprising five α-helices. These three repeats pack consecutively to form a long stalk-like shape. The Cul1 C-terminal domain (CTD) is composed of a four-helix bundle (4HB), an α/β domain and two copies of the winged-helix motif (WHA and WHB). The 4HB connects the N-terminal domain to CTD and organizes other subdomains in the CTD. It packs with the α/β domain and the long H29 helix, which connects WHA and WHB. The α/β domain and the N-terminal β-strand of Rbx1 form an intermolecular five-stranded β-sheet. One face of the WHB interacts with the long H29 helix and the 4HB, and the other contacts the RING domain of Rbx1. This compact architecture has been proposed to represent the 'closed' conformation of the Cul1–Rbx1 complex (Fig. 2a)^14^.

Although there is no high-resolution structure available for the Nedd8∼Cul1–Rbx1 complex, the crystal structure of Nedd8∼Cul5^CTD^–Rbx1 was previously resolved^14^. By threading the Cul1^CTD^ sequence into the neddylated Cul5 structure, we have derived a homology model of Nedd8∼Cul1–Rbx1 (Fig. 2b). Similar to the structure of Nedd8∼Cul5^CTD^–Rbx1 (ref. 14), this model shows that neddylation has minor effects on the structures of individual subdomains, but induces marked rearrangements in their relative positions. The H29 helix rotates about 45°, which changes the WHB position relative to the 4HB and α/β domain. The repositioning of WHB abolishes the interaction between the WHB and the Rbx1 RING domain and frees the latter from the Cul1 scaffold. Nedd8 contacts the WHB to stabilize this 'open' state of the Cul1–Rbx1 complex. Two orientations for the RING, resulting from crystal packing, are observed in the crystal structure of Nedd8∼Cul5^CTD^–Rbx1, indicating that the relative position of the RING domain and cullin scaffold are very promiscuous in solution. Therefore, we have generated two structural models of Nedd8∼Cul1–Rbx1 to describe the two different RING conformations (I and II) in the complex, and their overlays are illustrated in Fig. 2b. As shown, RING I (in yellow) is more proximal to the Cul1 scaffold, whereas RING II (in grey) is more distal. It is noted that the orientations of the Cul1 scaffold and Nedd8 remain the same in both RING conformations (Fig. 2b).

To further elucidate the structures of Cul1–Rbx1 complexes, we focused on the identification and quantification of interlinked peptides as they are most informative in describing residue proximity and interaction contacts in 3D structures. With our XL-MS strategy, we have identified a total of 68 unique interlinked d_0_/d_10_ peptide pairs from eight replicate sets of comparison experiments (Supplementary Table 1), representing 27 intraprotein and 17 interprotein linkages. To correlate our XL-MS data with the current structural models of Cul1–Rbx1 complexes, we first generated K–K linkage maps of Cul1–Rbx1 and Nedd8∼Cul1–Rbx1 based on the interlinks identified from each sample, as shown in Fig. 2c,d. It is important to note that our structural models of Nedd8∼Cul–Rbx1 with either RING (I) or RING (II) conformations have the same cross-link maps as shown in Fig. 2d. Interestingly, 23 of 25 intraprotein Cul1 K–K linkages are localized in Cul1^CTD^ regions that interact with Rbx1 and Nedd8. In addition, 5 and 10 linkages represent interprotein interactions of Cul1 with Rbx1 or Nedd8, respectively. Collectively, extensive interactions among the three proteins were detected for us to evaluate the structural differences between the various forms of Cul1–Rbx1 complexes.

Next, we have mapped the identified cross-linked residues onto the structural models of Cul1–Rbx1 and Nedd8∼Cul1–Rbx1, respectively, and calculated the distances between α-carbons (C_α_–C_α_ distance) of cross-linked lysines using the molecular visualization software PyMOL. Considering the lengths of the DMDSSO (11 Å) and lysine side chains as well as backbone dynamics, the theoretical upper limit for the C_α_–C_α_ distance between DMDSSO cross-linked lysine residues is ∼30 Å, suggesting that lysines within distance <30 Å can be preferably cross-linked by DMDSSO. To examine the distance constraints of identified cross-links, we have plotted the distance distribution of the Cul1–Rbx1 cross-link data set (Fig. 2e). Ninety per cent of cross-links satisfy the distance cutoff of 30 Å, indicating a good correlation with the current known structure of Cul1–Rbx1. However, when plotting Nedd8∼Cul1–Rbx1 cross-link data to either of our homology-derived models, only 64% of cross-links (23/36) are within the desired distance constraint (Fig. 2f). In fact, the cross-links outside the cutoff predominantly represent interactions among Nedd8, Rbx1 and the C-terminal domain of Cul1. As Rbx1 is suspected to be mobile in previous publications^14^, we then excluded 6 Rbx1-associated cross-links, thus yielding 30 remaining cross-links describing interactions within and between Nedd8 and Cul1 proteins. As a result, ∼73% of linkages (22/30) fall within our expected distance constraints (Fig. 2g), with the 8 outliers all representing cross-links that involve either Nedd8 or the winged-helix domains of Cul1^CTD^. This discrepancy could be explained by either the inaccuracy of the Nedd8∼Cul1–Rbx1 structural model in those regions or a highly dynamic topology associated with the 'open' conformation.

### Quantitation of DMDSSO cross-linked peptides

Generally, the likelihood of forming a cross-link between two given lysine residues is dependent on multiple factors. One of the important aspects is the 3D spatial distance between cross-linkable lysines. In addition, the relative orientations of proteins and their subdomains in different conformations under compared conditions can influence the relative reactivity of lysine residues. For instance, lysine residues localized in buried or protected regions would have decreased solvent and cross-linker accessibility compared with flexible, unprotected regions. Moreover, certain conformations could potentially influence the electronic environments of lysine residues by positioning them to form salt-bridge interactions with nearby acidic residues, decreasing their relative reactivity. Therefore, a combination of multiple factors could ultimately be responsible for the differences in observed spectral abundances of cross-linked peptides. Nonetheless, comparative analysis using QXL-MS strategies can unravel conformational changes of protein complexes under different conditions^24^,^38^. Of the total 68 unique interlinks identified in this work, 41 were identified at least in three biological replicates—our minimum requirement for reproducibility—representing 26 unique and high-confidence K–K linkages that were used for quantitative structural comparisons. Among them, there are two linkages associated with K720 of Cul1, which is the neddylation site and therefore covalently modified in the two neddylated Cul1–Rbx1 complexes, but free in unmodified Cul1–Rbx1 complex. As such, the two identified interlinked peptides associated with K720 of Cul1 were only detected in Cul1–Rbx1 complex, and were excluded from further analyses. The final list of 24 unique and quantifiable K–K linkages used for assessing structural changes of Cul1–Rbx1 complexes is summarized in Table 1. As shown, 13 were intraprotein (12 Cul1–Cul1 and 1 Rbx1–Rbx1) and 11 were interprotein (3 Cul1–Rbx1, 7 Cul1–Nedd8 and 1 Rbx1–Nedd8) interlinks. Among them, 15 linkages exhibited significant changes (≥4-fold) in their relative abundances and suggested structural differences in different samples, while the remaining 9 displayed marginal changes (<2-fold), indicating those interaction regions are relatively stable.

### Comparison of Cul1–Rbx1 and Nedd8∼Cul1–Rbx1 complexes

**Figure 3:**
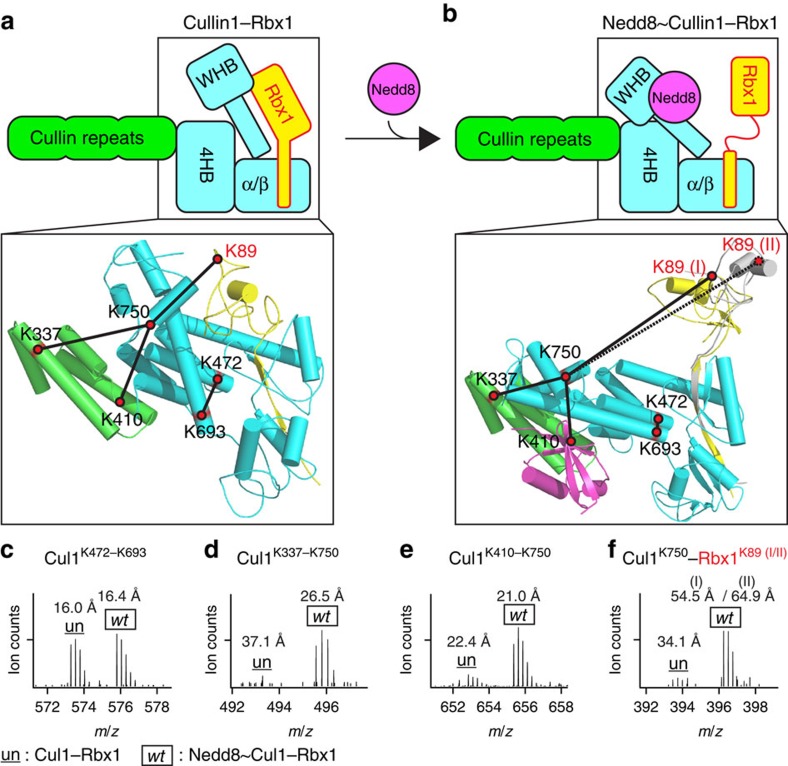
Quantitative analysis of K–K linkages to determine neddylation-dependent structural changes in the Cul1–Rbx1 complex. Structural representation of (**a**) unneddylated Cul1–Rbx1 in the 'closed' state, (**b**) neddylated Cul1–Rbx1 in the 'open' conformation, in which K89 (I) and K89 (II) represent K89 position in Rbx1 RING (I) (in yellow) or (II) (in grey) conformations, respectively. The insets display the mapping of four selected interlinks onto the structures of Cul1–Rbx1 complexes, whose MS^1^ spectra are displayed as follows: (**c**) Cul1^K472–K693^, (**d**) Cul1^K337–K750^; (**e**) Cul1^K410–K750^; (**f**) Cul1^K750^–Rbx1^K89(I/II)^. These d_0_/d_10_-DMDSSO cross-linked peptide pairs measured in MS^1^ were used to determine their relative abundance ratios between unneddylated and neddylated Cul1–Rbx1 complexes for quantitative analysis (Table 1).

Existing structural models have suggested that unneddylated Cul1–Rbx1 adopts a 'closed' conformation, while neddylated Cul1–Rbx1 exists in an 'open' state, as represented in Fig. 3a,b. To determine the structural effects of neddylation in the context of the full-length proteins, we examined intraprotein interlinks identified within Cul1 and Rbx1, respectively. For the 12 intraprotein interlinks identified within Cul1, 6 of them (that is, Cul1^K410–K743^, Cul1^K417–K689^, Cul1^K468–K693^, Cul1^K472–K689^, Cul1^K472–K693^ and Cul1^K701–K708^) exhibited below twofold difference between Cul1–Rbx1 and Nedd8∼Cul1–Rbx1 complexes, suggesting that there were no substantial structural reorientations between these cross-linked lysine residues upon neddylation (Table 1). In consistence with the cross-linking data, all of their C_α_–C_α_ distances are within 30 Å in the current Cul1–Rbx1 and Nedd8∼Cul1–Rbx1 complex models. For example, the relative spectral abundance ratio of the Cul1^K472–K693^ linkage in unneddylated and neddylated Cul1–Rbx1 is ∼1, and their respective C_α_–C_α_ distances are 16.0 and 16.4 Å (Fig. 3c). Although the 4HB and α/β domains containing these two residues become closer (Fig. 3a,b), the overall 3D spatial distance of these two residues has minimal change, thus leading to comparable cross-linking efficiency.

In contrast, the remaining six intraprotein interlinks of Cul1 had at least fourfold difference in their relative abundance ratios, in which five interlinks (that is, Cul1^K337–K750^, Cul1^K410–K750^, Cul1^K464–K693^, Cul1^K464–K743^ and Cul1^K693–K743^) were detected much more dominantly in Nedd8∼Cul1–Rbx1 complex and one (that is, Cul1^K431–K472^) significantly abundant in unneddylated Cul1–Rbx1 (Table 1; Fig. 3). These differences indicate that the two complexes feature substantial structural differences in regions containing the cross-linked lysines. In this study, two lysine residues that are proximal (<30 Å) would have higher chance of being captured by DMDSSO cross-linkers, which in turn increases the spectral abundance compared with one between lysine residues that are spatially distant (>30 Å). Figure 3d displays the MS spectrum of the Cul1^K337–K750^ interlink, and quantitative analysis revealed that this interaction occurs much more favourably in Nedd8∼Cul1–Rbx1 than Cul1–Rbx1, on an average of 20:1. From the current models, the C_α_–C_α_ distances between K337 and K750 of Cul1 were calculated to be 36.3 and 26.5 Å based on the Cul1–Rbx1 structure and our Nedd8∼Cul1–Rbx1 model, respectively. These calculated C_α_–C_α_ distances fall outside of and within the distance that can be cross-linked by DMDSSO, which are in agreement with the increased spectral abundance of this interlink in Nedd8∼Cul1–Rbx1 compared with Cul1–Rbx1.

Similarly, the intraprotein interlink Cul1^K410–K750^ was calculated to be on an average of approximately seven times more abundant in wild-type neddylated than unneddylated forms (Table 1; Fig. 3e). However, distinct from the Cul1^K337–K750^ interlink, the C_α_–C_α_ distances of K410 and K750 in the current models were determined to be 22.4 and 21.0 Å, respectively. Despite this similarity in their calculated proximities, the differential spectral abundance suggests that their cross-link is obstructed in unneddylated Cul1. In addition to distance, cross-linked peptide spectral abundance is influenced by the relative orientation of the lysine pair and their surroundings. In fact, K410 and K750 of unneddylated Cul1 point away from each other and are separated by other residues, which can presumably impede the cross-linking reaction. Similarly, an additional four Cul1 intraprotein interlinks (that is, Cul1^K464–K693^, Cul1^K464–K743^, Cul1^K693–K743^ and Cul1^K431–K472^) have no apparent correlation between their relative spectral abundance ratios and respective C_α_–C_α_ distance (<30 Å). However, most of them can be rationalized based on the structural environment of the lysine residues in the context of the current structure models (Supplementary Fig. 3). The Cul1^K693–K743^ interlink represents the only noticeable outlier, which is >30-folds more abundant in Nedd8∼Cul1–Rbx1 than Cul1–Rbx1, albeit a similar C_α_–C_α_ distance in the two models. K693 and K743 are located on the H29 helix and the WHB domain, which together act as a single rigid body. Their preferred cross-links in the neddylated Cul1–Rbx1 cannot be explained without significant changes of the current model of Nedd8∼Cul1–Rbx1. Overall, our results suggest that certain regions in the Cul1 scaffold, including the structural elements where those lysines are located, likely undergo profound structural reorientations in response to neddylation.

**Figure 4:**
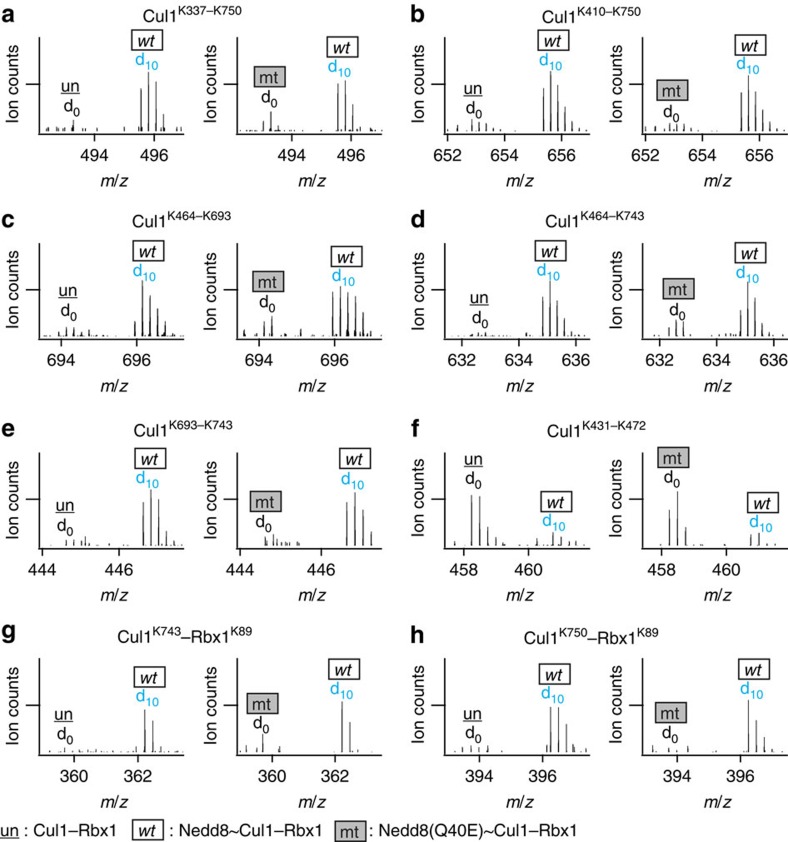
Deciphering the structural dynamics of Cul1–Rbx1 complexes using QXL-MS. Eight selected K–K linkages are presented to describe conformational changes in the three types of Cul1–Rbx1 complexes. Two sets of pairwise comparison results (that is, un (d_0_) versus wt (d_10_) and mt (d_0_) versus wt (d_10_)) are displayed for each selected cross-link, in which both the un and mt forms were cross-linked by d_0_-DMDSSO and the wt form was cross-linked by d_10_-DMDSSO. MS^1^ spectra of (**a**) Cul1^K337–K750^, (**b**) Cul1^K410–K750^, (**c**) Cul1^K464–K693^, (**d**) Cul1^K464–K743^, (**e**) Cul1^K693–K743^, (**f**) Cul1^K431–K472^, (**g**) Cul1^K743^–Rbx1^K89^ and (**h**) Cul1^K750^–Rbx1^K89^. The sequences of these cross-links are summarized in Supplementary Table 1. The relative spectral abundance of cross-links (d_0_:d_10_) measured during MS analysis describes the cross-linkability of lysine residues in 3D structure.

To further dissect the impact of neddylation, we examined the three unique Cul1–Rbx1 interprotein K–K linkages identified here (Table 1; Figs 3 and 4). Two cross-links, Cul1^K743^–Rbx1^K89^ and Cul1^K750^–Rbx1^K89^, were quantitatively determined to have spectral abundances on an average of 10-fold higher in neddylated Cul1–Rbx1 compared with their unneddylated counterparts. Mapping of Cul^K743^–Rbx1^K89^ and Cul^K750^–Rbx1^K89^ to the Cul1–Rbx1 structure determines their C_α_–C_α_ distances to be 31.9 and 34.1 Å, respectively, just outside the range covered by DMDSSO and accounting for their low cross-linking abundances. However, when mapped to our homology-derived models of neddylated Cul1–Rbx1 with either RING (I) or RING (II) conformations, those same interlinks yielded C_α_–C_α_ distances of 59.2 Å (I)/66.0 Å (II) and 57.5 Å (I)/64.9 Å (II), respectively (Table 1; Fig. 3f), even more unlikely to be cross-linked by DMDSSO. Instead, these unusual cross-links must be explained by either the structural flexibility of the 'open-state' conformation exhibited by Nedd8∼Cul1–Rbx1 or a geometry different from the current model. In the crystal structure of Nedd8∼Cul5^CTD^–Rbx1, neddylation causes the globular RING domain of Rbx1 to eject from the WHB while remaining tethered to the Cul5^CTD^ by its N-terminal sequence. As a result, Rbx1 is free to sample the 3D space above Cul5^CTD^. Our observations on the three Cul1–Rbx1 interprotein interlinks indicate that such a dynamic topology is plausible and can account for the formation of linkages with lysine residues that are too distant to be cross-linked in the unneddylated complex.

Interestingly, mapping of the identified K-K linkages between Cul1 and Nedd8 to the homology-derived Nedd8∼Cul1–Rbx1 model shows that three out of seven cross-linking events were calculated to bridge C_α_–C_α_ distances >30 Å, with two more above 28.5 Å (Table 1). This suggests that the position of Nedd8 relative to 4HB and α/β subdomains of Cul1, which comprise the majority of the Cul1–Nedd8 interlinks, may not be accurate in the current model. On one hand, the crystal structure of the Nedd8∼Cul5^CTD^–Rbx1 complex, which our Nedd8∼Cul1–Rbx1 model was based on, might represent snapshots of an otherwise dynamic scaffold in addition to the flexibly linked Rbx1 RING domain. On the other hand, it remains possible that neddylation may cause different conformational changes on different cullins. Therefore, a more accurate structure model of the Nedd8∼Cul1–Rbx1 complex is needed to explain all comparative cross-links between the free and modified Cul1–Rbx1 assembly.

### Effects of Nedd8 deamidation on Cul1–Rbx1

To investigate the structural mechanism underlying deamidation of Nedd8, we conducted pairwise structural comparisons between Nedd8∼Cul1–Rbx1 (wt) and Nedd8(Q40E)∼Cul1–Rbx1 (mt) complexes using the same DMDSSO-based QXL-MS strategy as described above. Similar to the previous results obtained from the comparison between unneddylated and wild-type neddylated Cul1–Rbx1 complexes, the six Cul1 interlinks (that is, Cul1^K410–K743^, Cul1^K417–K689^, Cul1^K468–K693^, Cul1^K472–K689^, Cul1^K472–K693^ and Cul1^K701–K708^) and one Rbx1 interlink (that is, Rbx1^K19–K25^) displayed non-significant changes (<2-fold) when comparing their spectral abundances in wt-neddylated and mt-neddylated∼Cul1–Rbx1 complexes (Table 1). Interestingly, the five interlinks of Cul1 (that is, Cul1^K337–K750^, Cul1^K410–K750^, Cul1^K464–K693^, Cul1^K464–K743^ and Cul1^K693–K743^) that were found primarily in Nedd8∼Cul1–Rbx1 compared with unneddylated Cul1–Rbx1 were also difficult to detect in Nedd8(Q40E)∼Cul1–Rbx1 (Fig. 4a–e). Furthermore, the Cul1^K431–K472^ interlink, which was found to be much more abundant in unneddylated Cul1–Rbx1 than Nedd8∼Cul1–Rbx1, was also detected more intensely in Nedd8 (Q40E)∼Cul1–Rbx1 (Fig. 4f). Overall, the relative abundance ratios of these core CRL interlinks are similar when comparing Nedd8∼Cul1–Rbx1 to both Cul1–Rbx1 and Nedd8(Q40E)∼Cul1–Rbx1, respectively, suggesting that covalent attachment of Nedd8(Q40E) to Cul1 did not result in the same conformational changes in Cul1 as the wild-type Nedd8 modification.

This observation is also supported by the identification of interlinked peptides between Cul1 and Rbx1. The Cul1^K743^–Rbx1^K89^ and Cul1^K750^–Rbx1^K89^ interprotein interlinks were primarily detected in Nedd8∼Cul1–Rbx1, but not in Cul1–Rbx1 or Nedd8(Q40E)∼Cul1–Rbx1 (Fig. 4g,h). Collectively, the quantitative MS profiles of the identified Nedd8(Q40E)∼Cul1–Rbx1 linkages are much more similar to those of unneddylated Cul1–Rbx1.

**Figure 5:**
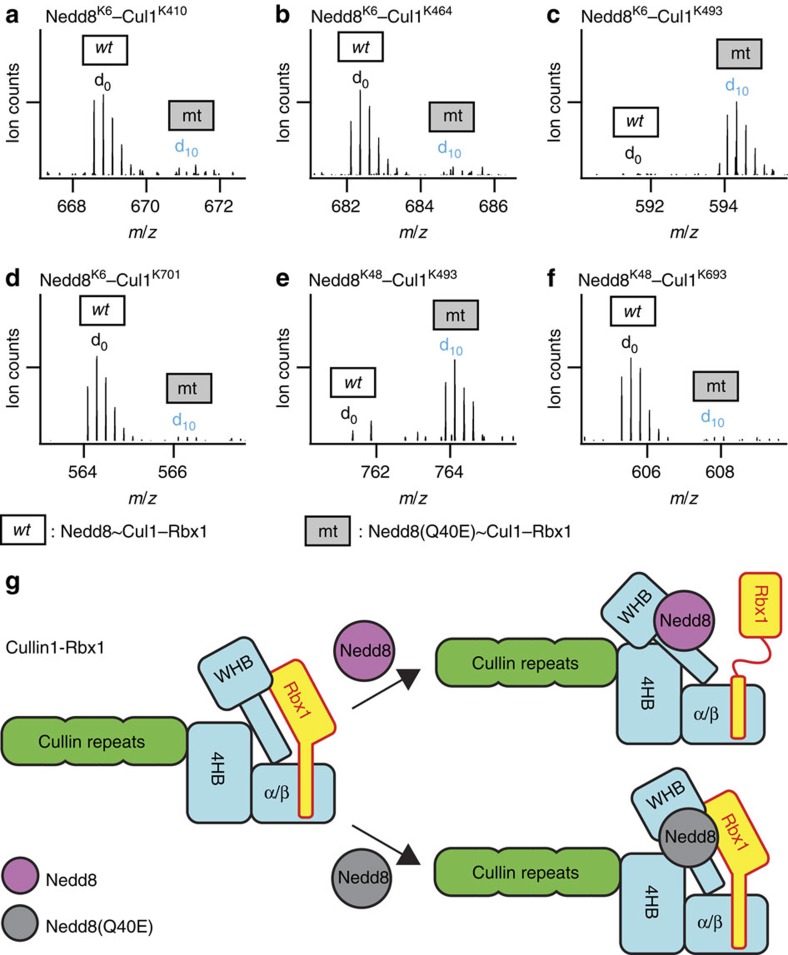
Elucidation of structural dissimilarities between wt- and mt-neddylated Cul1–Rbx1 complexes by quantitative analysis of Cul1–Nedd8 interlinks. MS1 spectra of (**a**) Nedd8^K6^–Cul1^K410^, (**b**) Nedd8^K6^–Cul1^K464^, (**c**) Nedd8^K6^–Cul1^K493^, (**d**) Nedd8^K6^–Cul1^K701^, (**e**) Nedd8^K48^–Cul1^K493^ and (**f**) Nedd8^K48^–Cul1^K693^. The mt form was d_0_-DMDSSO cross-linked, and the wt form was d_10_-DMDSSO cross-linked. (**g**) Proposed models for Nedd8-dependent conformational changes of the Cul1–Rbx1 complex.

The structural dissimilarities in the two types of neddylated Cul1–Rbx1 complexes are further confirmed by Cul1–Nedd8 linkage comparisons. We have identified seven unique interprotein K–K linkages between Cul1 and Nedd8 as summarized in Table 1. Interestingly, all of the Cul1–Nedd8 intersubunit interlinks had relative abundance ratios indicating significant differences (≥4-fold) between Nedd8∼Cul1–Rbx1 and Nedd8(Q40E)∼Cul1–Rbx1 (Fig. 5). Among them, five cross-links (Nedd8^K6^–Cul1^K410^, Nedd8^K6^–Cul1^K464^, Nedd8^K6^–Cul1^K468^, Nedd8^K48^–Cul1^K693^ and Nedd8^K6^–Cul1^K701^) were only detected in Nedd8∼Cul1–Rbx1, while the remaining cross-links (Nedd8^K6^–Cul1^K493^ and Nedd8^K48^–Cul1^K493^) were only measured in Nedd8(Q40E)∼Cul1–Rbx1. In particular, K6 of Nedd8 and K493 of Cul1 are localized to opposite sides of the Cul1 scaffold in the Nedd8∼Cul1–Rbx1 model, resulting in a C_α_–C_α_ distance >30 Å. Therefore, this Nedd8^K6^–Cul1^K493^ cross-link preferably detected in Nedd8(Q40E)∼Cul1–Rbx1 further suggests that the Q40E mutation imparts a large degree of influence on the position of Nedd8 in relation to the Cul1 scaffold. Taken together, our results have demonstrated that Nedd8(Q40E) cannot induce the same structural effect on Cul1–Rbx1 as wild-type Nedd8, and the overall conformation of Nedd8(Q40E)∼Cul1–Rbx1 is much more similar to that of unneddylated Cul1–Rbx1 (Fig. 5a–f).

## Discussion

We have developed an effective QXL-MS workflow based on our previously developed pair of isotope-labelled (that is, d_0_ and d_10_) MS-cleavable DMDSSO^31^ to characterize the structural differences and similarities of three Cul1–Rbx1 complexes. This approach allows us to quantitatively assess neddylation-dependent conformational changes within the Cul1–Rbx1 complex and gain insights into the molecular basis underlying its activation mechanism. In this work, we have demonstrated that DMDSSO reagents are well suited to quantitatively compare protein complexes as they cross-link proteins with similar efficiency and the relative spectral intensity ratios of d_0_- and d_10_-DMDSSO cross-linked peptides are indicative of their respective abundances in the two compared samples. Thus, these isotope-coded cross-linkers can be used orthogonally to study differential protein structures by characterizing their intraprotein and interprotein interlinked peptides to describe protein interactions associated with conformational changes.

With this QXL-MS approach, we have reproducibly quantified 24 unique intraprotein and interprotein lysine–lysine linkages within the three different forms of Cul1–Rbx1 complexes (that is, Cul1–Rbx1, Nedd8∼Cul1–Rbx1 and Nedd8(Q40E)∼Cul1–Rbx1). Although a substantial amount of cross-link data correlates well with existing models, several cross-links have spatial distances outside the desired range and cannot be rationalized based on current structure models. While our results generally support the homology model of Nedd8∼Cul1–Rbx1 derived from the Nedd8∼Cul5^CTD^–Rbx1 crystal structure^14^, a more accurate description of neddylation-induced conformational changes of the Cul1 scaffold calls for the necessity of a better defined model.

Independent of such a model, multiple pairwise comparisons have revealed that the molecular structure of Nedd8(Q40E)-modified Cul1–Rbx1 is very similar to that of its unmodified form, but significantly different from wild-type Nedd8-modified Cul1–Rbx1, indicating that Gln40 in Nedd8 is critical for the structural stability of neddylated Cul1–Rbx1. In the structure of Nedd8–Cul5^CTD^–Rbx1, Gln40 is proximal to the isopeptide bond between Nedd8 and Cul5 (ref. 14) and may interact with the cullin scaffold to stabilize its active conformation. On the other hand, the unmodified CRL catalytic core adopts a rigid, thermodynamically stable 'closed' structure, lacking ligase activity for polyubiquitination of substrates. During neddylation, CRL interacts with neddylation machinery and shifts to a flexible, 'open' conformation with the extending RING-finger domain^14^,^39^. The neddylated CRL remains in its active state until Nedd8 is removed (deneddylation). We propose that Gln40 in Nedd8 can interact with amino-acid residues in cullin through weak interactions, such as hydrogen bonds and electrostatic interactions, which are responsible for stabilization of the 'open' state. Deamidation of Gln40 abolishes or weakens these interactions such that the CRL switches back to its thermodynamically more stable 'closed' state. On the basis of our cross-link data, we have proposed schematic models representing neddylation-dependent conformational changes in the Cul1–Rbx1 complex by wild-type or mutant Nedd8 (Fig. 5g). As illustrated, wt-neddylation leads to the 'open' conformation of the CRL core in which Rbx1 is free to rotate as previously shown by crystallography^14^. In contrast, mt-neddylation of Cul1 prevents switching from the inactive to active state by maintaining the 'closed' structure of the CRL with Rbx1 embedded. To verify this hypothesis, an experimental structure of neddylated full-length CRL will be required.

In summary, we have successfully applied our recently developed MS-cleavable, stable isotope-labelled cross-linkers d_0_-DMDSSO and d_10_-DMDSSO to quantitatively study structural differences in Cul1–Rbx1 complexes in response to neddylation. Such structural characterization has previously been hindered using conventional structural tools because of their large sizes (over 100 kDa) and dynamic conformations. Our QXL-MS approach enables us to quantitatively compare multiple lysine interlinks in three types of full-length Cul1–Rbx1 complexes. Comparing these cross-linkage profiles, we found that neddylation can induce large structural rearrangements of the Cul1–Rbx1 complex, which are partially consistent with structural models obtained with truncated and neddylated Cul5–Rbx1 complex. Our results also indicates Nedd8(Q40E)-conjugated Cul1–Rbx1 has a similar structure as that of free Cul1–Rbx1, answering the puzzle of how a subtle change of a single Nedd8 amino acid, Gln^40^, can abolish the activity of the much larger CRL complex. Given the speed and accuracy of the approach, we expect that our QXL-MS strategy will enable us to perform future studies in characterizing E2–E3 interactions and further dissect the action mechanism of CRLs during protein ubiquitination. In addition, our work has paved the way for adapting QXL-MS methods for elucidating dynamic structures of proteins and protein complexes in the future.

## Methods

### Materials and reagents

General chemicals were purchased from Fisher Scientific or VWR international. Sequencing grade-modified trypsin was purchased from Promega (Fitchburg, WI).

### Preparation of Cul1–Rbx1 protein complexes

The heterodimeric NEDD8-activating enzyme APPBP1–Uba3 was prepared similarly as before^40^. Briefly, APPBP1 was subcloned into a modified pGEX4T1 (Amersham Biosciences) vector containing a glutathione *S*-transferase (GST) tag followed by a Tobacco etch virus (TEV) protease cleavage site, while Uba3 was subcloned into a modified pET15b (Novagen) vector containing a chloramphenicol resistance cassette. GST–APPBP1 and Uba3 were co-expressed in BL21(DE3) (Novagen) and purified by glutathione-affinity chromatography. After TEV cleavage, the APPBP1–Uba3 complex was further purified by anion exchange and gel filtration.

Nedd8 and the Nedd8-conjugating enzyme Ubc12 were subcloned into the same pGEX4T1 vector. Both were expressed in *E. coli* BL-21(DE3) cells and purified by glutathione-affinity and anion-exchange chromatography. In this study, we used a truncated version of Nedd8 ending at glycine 76, representing its mature form.

Two short unstructured segments in the N-terminus of Cul1 (residues 1–12 and 58–81) were removed from full-length human Cul1 to form Cul1^ΔN^ (referred to here as Cul1). Both Cul1 and Rbx1^16–108^ were fused with an N-terminal His_6_ tag followed by a TEV cleavage site and co-expressed in BL-21(DE3). The complex was first purified by a Ni^2+^ sepharose-affinity column (GE Healthcare) and further purified by cation-exchange and gel filtration chromatography after TEV cleavage. To prepare Nedd8∼Cul1–Rbx1, 10 μM purified Cul1–Rbx1 was neddylated with 10 μM GST–Nedd8 in the presence of 0.2 μM APPBP1–Uba3 and 0.5 μM Ubc12 for 1 h at 4 °C. Nedd8∼Cul1–Rbx1 was then separated from free Cul1–Rbx1 by a glutathione-affinity column. After TEV cleavage, Nedd8∼Cul1–Rbx1 was eluted off the column and further purified by cation-exchange and gel filtration chromatography. Nedd8(Q40E)-modified Cul1–Rbx1 was purified similarly to the wild type.

Full-length human ubiquitin-activating enzyme Ube1 was expressed as a GST fusion protein in High Five insect cells using the Bac-to-Bac baculovirus expression system (Invitrogen). Insect cells were collected 48–72 h post infection and lysed, followed by glutathione-affinity chromatography. Recombinant human Cdc34 was overexpressed and purified from *E. coli* by a similar approach as the Nedd8 purification. Recombinant untagged ubiquitin (Ub) was expressed in BL21(DE3). After sonication and centrifugation, cleared lysate was adjusted to 3.5% perchloric acid. After precipitated protein was removed by centrifugation, ubiquitin in the supernatant was further purified by cation-exchange chromatography and dialysis against 20 mM Tris, pH 8.0 thoroughly.

### Ubiquitination assays

For free ubiquitin chain synthesis assay, a mixture containing 100 μM Ub, 0.3 μM UBE1 and 1.0 μM Cdc34 was incubated with 0.4 μM Cul1–Rbx1 variants in a reaction buffer of 50 mM Tris-HCl, 200 mM NaCl, 2 mM ATP and 10 mM MgCl_2_, pH8.0. After incubation at 37 °C for 4 h, the reaction mixtures were resolved by a 15% SDS–PAGE gel and transferred onto a nitrocellulose membrane, which was incubated overnight with a mouse monoclonal anti-ubiquitin antibody (Sigma-Aldrich, #U0508) at 1:2,500 dilution. The membrane was washed and incubated with horseradish peroxidase-linked ECL-anti mouse IgG (GE Healthcare, #NA931V) for 1 h. Free ubiquitin chains were visualized using SuperSignal West Pico Chemiluminescent Substrate (Pierce Biotechnology, #34080).

For the CRY2 ubiquitination assay, a reaction mixture containing 0.2 μM CRY2–FBXL3–SKP1 complex^36^, 70 μM Ub, 0.15 μM UBE1 and 1.5 μM Cdc34 was incubated with 0.4 μM Cul1–Rbx1 variants in a reaction buffer of 40 mM Tris-HCl (pH 7.5), 2 mM dithiothreitol, 5 mM MgCl_2_ and 2 mM ATP. The reactions were carried out at 37 °C and quenched at different time points by adding SDS–PAGE loading buffer, then analysed by western blot with a rabbit anti-CRY2 antibody(LifeSpan BioSciences, Inc. #LS-C6229) at 1:1,000 dilution and horseradish peroxidase-linked ECL-anti rabbit IgG (GE Healthcare, #NA934V).

### DMDSSO cross-linking and digestion of Cul1–Rbx1 complexes

Purified complexes were diluted to 4 μM in 20 mM HEPES (pH 7.5) and reacted with d_0_- or d_10_-DMDSSO in a molar ratio of 1:25 (protein: cross-linker) for 45 min at room temperature and quenched with excess ammonium bicarbonate. Cross-linked proteins were then separated by SDS–PAGE and visualized by Coomassie blue. Bands corresponding to cross-linked complexes were excised, reduced with tris (2-carboxyethyl) phosphine for 30 min at room temperature and alkylated with chloroacetamide for 30 min at room temperature in dark, and then digested with trypsin at 37 °C overnight. Peptide digests were extracted, concentrated and reconstituted in 3% ACN/2% formic acid before LC-MS^*n*^ analysis. To allow quantitative pairwise complex comparisons, individually cross-linked proteins were strategically mixed, for example, d_0_-DMDSSO cross-linked Cul1–Rbx1 with d_10_-DMDSSO cross-linked Nedd8∼Cul1–Rbx1, at a 1:1 ratio and subjected to subsequent analysis together as outlined above.

### Liquid chromatography-multistage tandem mass spectrometry

DMDSSO cross-linked peptides were analysed by LC-MS^*n*^ utilizing an LTQ-Orbitrap XL MS (Thermo Fisher, San Jose, CA) coupled on-line with an Easy-nLC 1,000 (Thermo Fisher, San Jose, CA) as previously described^29^,^31^. Each MS^*n*^ experiment consists of one MS scan in FT mode (350–1,400 m/z, resolution of 60,000 at m/z 400) followed by two data-dependent MS2 scans in FT mode (resolution of 7,500) with normalized collision energy at 20% on the top two MS peaks with charges at 3+ or up, and three MS3 scans in the LTQ with normalized collision energy at 35% on the top three peaks from each MS2.

### Data analysis, identification and quantification of cross-linked peptides

Monoisotopic masses of parent ions and corresponding fragment ions, parent ion charge states, and ion intensities from LC-MS^2^ and LC-MS^3^ spectra were extracted using an in-house software based on the Raw_Extract script from Xcalibur v2.4 (Thermo Scientific)^29^,^30^,^31^. MS^3^ data were subjected to a developmental version of Protein Prospector (v. 5.10.10) for database searching, using Batch-Tag against a limited database containing recombinant Cul1, Rbx1, Nedd8 and Nedd8(Q40E) sequences with mass tolerances for parent ions and fragment ions set as ±20 p.p.m. and 0.6 Da, respectively. Trypsin was set as the enzyme with five maximum missed cleavages allowed. A maximum of five variable modifications were also allowed, including protein N-terminal acetylation, methionine oxidation, N-terminal conversion of glutamine to pyroglutamic acid, asparagine deamidation and cysteine carbamidomethylation. In addition, three defined modifications on uncleaved lysines and free protein N-termini were also selected: alkene (A: C_4_H_4_O, +68 Da; or A*: C_4_H_−1_D_5_O, +73 Da), sulfenic acid (S: C_4_H_6_O_2_S, +118 Da; or S*: C_4_H_1_D_5_O_2_S, +123 Da) and unsaturated thiol (T: C_4_H_4_OS, +100 Da; or T*: C_4_H_−1_D_5_OS, +105 Da) modifications, due to remnant moieties of d_0_- (that is, A, S and T) or d_10_-DMDSSO (that is, A*, S* and T*), respectively. It is noted that the sulfenic acid moiety often undergoes dehydration to become a more stable and dominant unsaturated thiol moiety (that is, T, +100 Da or T*, +105 Da) as previously described^29^,^30^,^31^. Initial acceptance criteria for peptide identification required a reported expectation value ≤0.1.

Integration of MS^*n*^ data was carried out using the in-house program LinkHunter, a revised version of the previously written Link-Finder program, to validate and summarize cross-linked peptides^22^,^29^. Basically, monoisotopic masses of parent ions measured in MS^1^ scans for those putative interlinked peptides are required to match the sum of the two MS^2^ cross-linked fragment ions that have been sequenced in MS^3^.

Only the identified interlinked DMDSSO cross-linked peptides were subjected for subsequent manual quantitation as only interlinked peptides provide the most useful information on protein structures. Using Skyline (v. 2.5.06157; https://skyline.gs.washington.edu), we have determined the spectral abundances of all individually identified cross-linked peptides in each pairwise comparison, and the calculated relative abundance of d_0_/d_10_ cross-linked peptides. This allows determining the relative occurrence of the identified K–K linkages across all purified complexes. All linkages were then mapped onto existing Cul1–Rbx1 crystal structure (PDB: 1LDJ), as well as the derived homology model of Nedd8∼Cul1–Rbx1 (ref. 32), to compare experimentally derived ratios of occurrence of K–K linkages to the C_α_–C_α_ distances as determined by structural models.

## Additional information

**How to cite this article:** Yu, C. *et al*. Gln40 deamidation blocks structural reconfiguration and activation of SCF ubiquitin ligase complex by Nedd8. *Nat. Commun*. 6:10053 doi: 10.1038/ncomms10053 (2015).

## Supplementary Material

Supplementary Information Supplementary Figures 1-3 and Supplementary Table 1

## Figures and Tables

**Table 1:** Comparative linkage profiles for SCF core ligases as determined by LC-MS^*n*^.

Linkages identified						Mapped distances (Å)		Quantitative ratio*		
Linkage	Cul1	Cul1	Rbx1	Rbx1	Nedd8	Cul1–Rbx1	wtNedd8∼Cul1–Rbx1	Cul1–Rbx1	wtNedd8∼Cul1–Rbx1	mtNedd8∼Cul1–Rbx1
**Cul1—Cul1**	K337	K750				37.1	26.5	0.05	1.00	0.20
	K410	K720				38.1	21.7	1.00	0.01	0.01
	K410	K743				17.4	22.5	0.85	0.67	1.00
	K410	K750				24.9	21.0	0.14	1.00	0.36
	K417	K689				18.8	24.8	1.00	0.60	0.91
	K431	K472				9.7	9.3	1.00	0.24	0.50
	K464	K693				15.8	11.9	0.36	1.00	0.41
	K464	K743				15.7	26.3	0.05	1.00	0.22
	K468	K693				13.7	12.2	1.00	0.49	0.85
	K472	K689				16.3	10.7	1.00	0.87	0.90
	K472	K693				15.7	16.4	0.94	0.87	1.00
	K693	K743				24.8	26.6	0.03	1.00	0.17
	K701	K708				11.1	10.5	0.69	1.00	0.92
**Cul1–Rbx1**†	K493		K89			30.2	40.8 (I)/55.2 (II)	1.00	0.67	0.77
	K720		K89			11.7	72.8 (I)/82.8 (II)	1.00	0.00	0.00
	K743		K89			26.8	59.2 (I)/66.0 (II)	0.06	1.00	0.10
	K750		K89			22.6	54.5 (I)/64.9 (II)	0.11	1.00	0.05
**Rbx1–Rbx1**			K19	K25		18.1	**---**	1.00	0.62	0.71
**Cul1–Nedd8**	K410				K6	—	17.0	0.01	1.00	0.22
	K464				K6	—	30.6	0.01	1.00	0.12
	K468				K6	—	35.3	0.05	1.00	0.23
	K493				K6	—	41.0	0.04	0.07	1.00
	K493				K48	—	29.9	0.01	0.11	1.00
	K693				K6	—	28.7	0.00	1.00	0.11
	K701				K6	—	19.6	0.01	1.00	0.13
**Rbx1†–Nedd8**			K89		K48	—	65.0 (I)/74.3 (II)	0.02	0.96	1.00

LC-MS^*n*^, liquid chromatography-multistage tandem mass spectrometry; SCF, Skp1–Cul1–F-box protein.

— Denotes distance undeterminable due to missing residues in structure/model.

^*^Spectral abundances normalized to the highest value for each linkage (per row).

^†^Mapped distances calculated for both Rbx1 RING conformation I and II (Fig. 2b).
