# Supplementary material for: Gln40 deamidation blocks structural reconfiguration and activation of SCF ubiquitin ligase complex by Nedd8
Source: Nat Commun. 2015 Dec 3;6:10053. doi: 10.1038/ncomms10053 (PMC4686759; doi:10.1038/ncomms10053)
Supplement: Supplementary Information — Supplementary Figures 1-3 and Supplementary Table 1 [file ncomms10053-s1.pdf]

Supplementary Figure 1

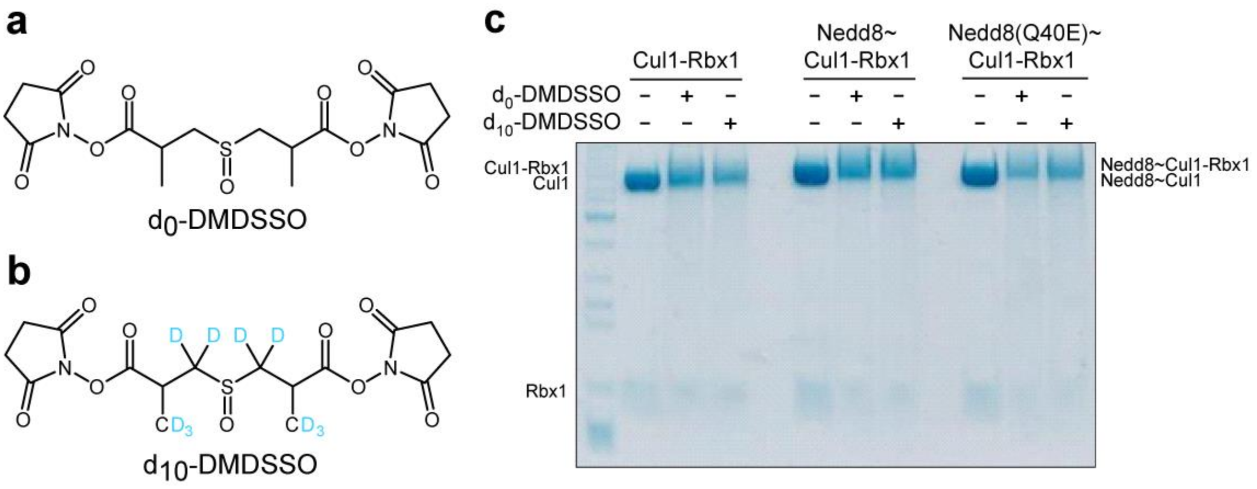

Supplementary Figure 1: d_0- and d_{10}-DMDSSO structure and cross-linking efficiency. Molecular structures of **(a)** d_0-DMDSSO and **(b)** d_{10}-DMDSSO. **(c)** SDS-PAGE separation of unneddylated, wild-type neddylated, and mutant Q40E neddylated Cul1-Rbx1 complexes cross-linked by d_0- and d_{10}-DMDSSO.

Supplementary Figure 2

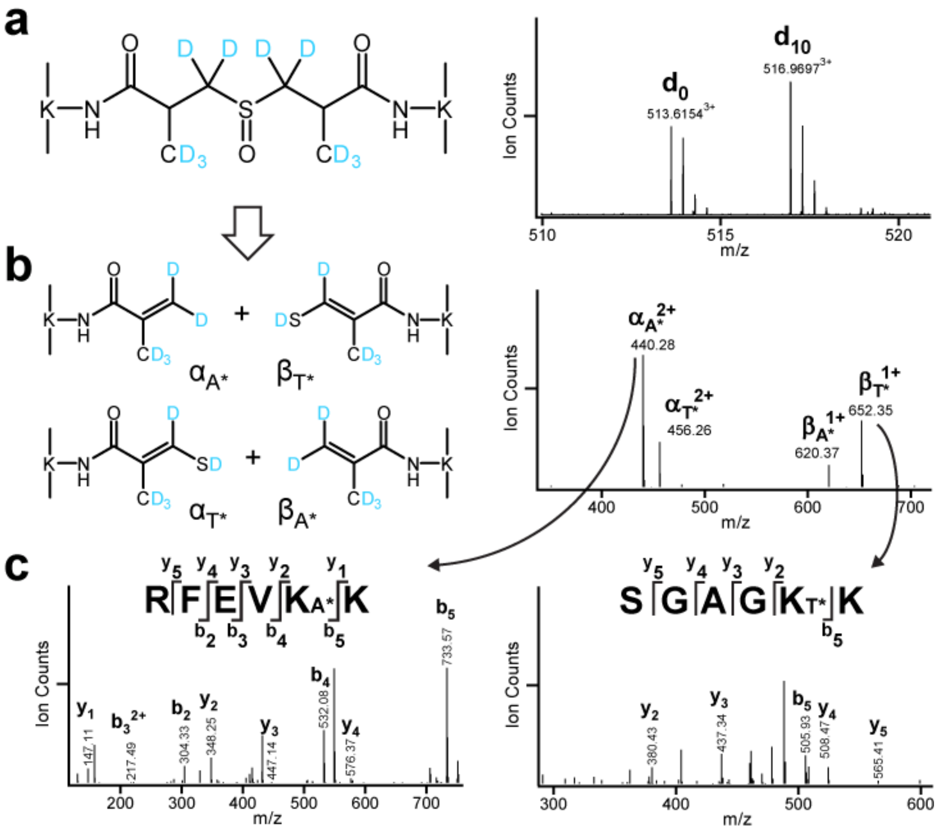

Supplementary Figure 2: MS^n analysis of d$_{10}$-DMDSSO inter-linked peptides. (a) MS^1 spectrum shows the detection of a pair of d$_0$-DMDSSO and d$_{10}$-DMDSSO cross-linked peptides (m/z 513.6154^{3+} and m/z 516.9697^{3+}), whose spectral relative abundance ratio is used for quantitation. (**b**) MS^2 analysis of the d$_{10}$-DMDSSO inter-linked peptides α-β (m/z 516.9697^{3+}) yielded two peptide fragment pairs: α_{A*}/β_{T*} (m/z 440.28^{2+}/652.35^{1+}) and α_{T*}/β_{A*} (m/z 456.26^{2+}/620.37^{1+}), confirming its cross-link type as an interlink. Subsequent MS^3 analyses of the α_{A*} (m/z 440.28^{2+}) and β_{T*} (652.35^{1+}) ions produced series of y and b ions which enabled unambiguous identification of α_{A*} as RFEVK$_{A*}$K of Rbx1 and β_{T*} as SGAGK$_{T*}$K of Rbx1. Integration of the MS^n (i.e. MS^1, MS^2 and MS^3) data has confirmed the d$_{10}$-DMDSSO cross-linked peptide as an intra-subunit interlink between K19 and K25 of Rbx1. K$_{A*}$: d$_{10}$-DMDSSO alkene modified lysine; K$_{T*}$: d$_{10}$-DMDSSO unsaturated thiol modified lysine.

Supplementary Figure 3

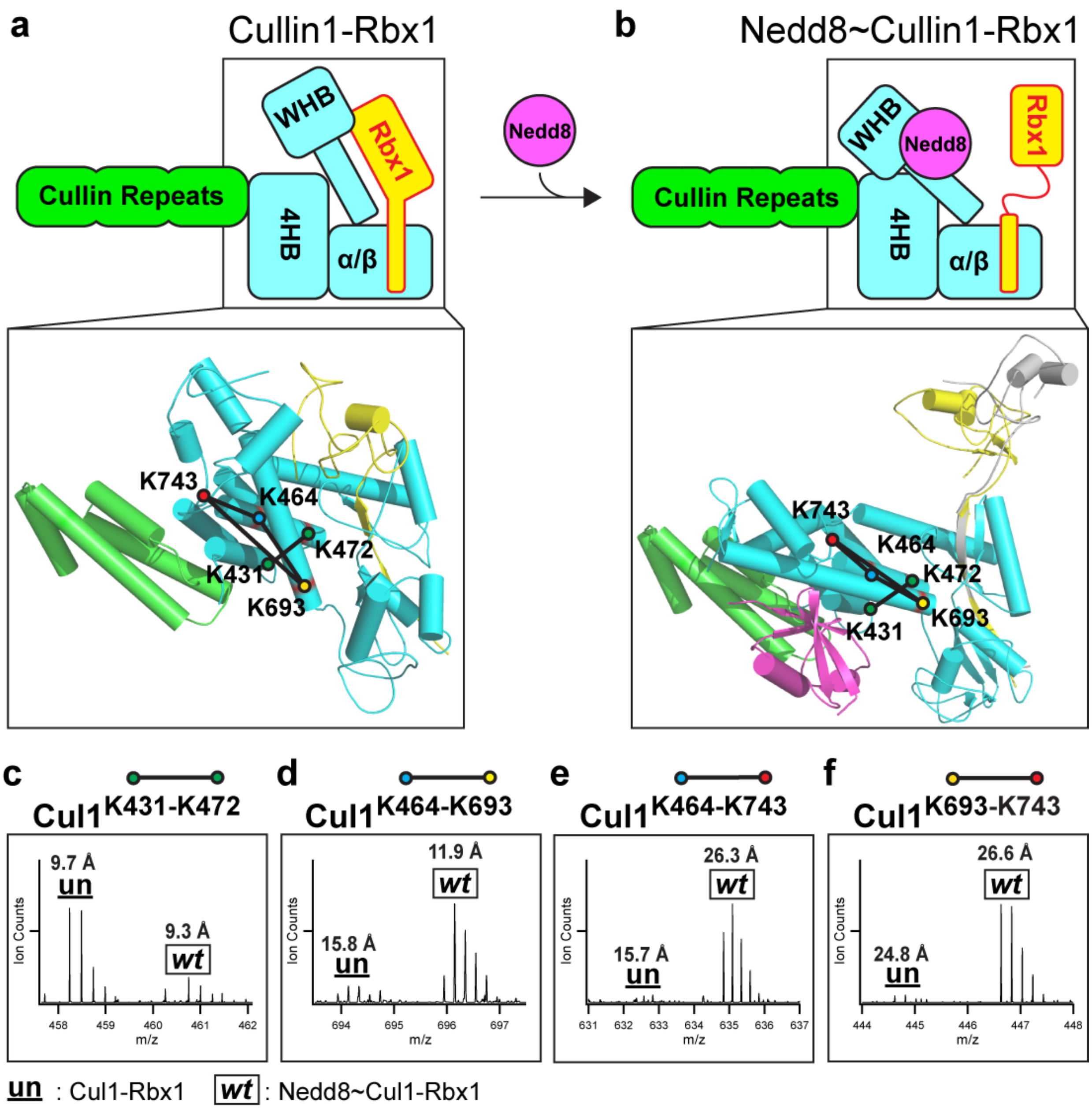

Supplementary Figure 3: Quantitative analysis of K-K linkages to determine neddylation-dependent structural changes in the Cul1-Rbx1 complex. Structural representation of (**a**) unneddylated Cul1-Rbx1 in the "closed" state, (**b**) neddylated Cul1-Rbx1 in the "open" conformation. The insets display the mapping of four selected inter-links onto the structures of Cul1-Rbx1 complexes, whose MS1 spectra are displayed as follows: (**c**) Cul1^{K43}1-Cul1^{K472}, (**d**) Cul1^{K464}-Cul1^{K693}, (**e**) Cul1^{K464}-Cul1^{K743}, and (**f**) Cul1^{K693}-Cul1^{K743}. These d_0/d_{10}-DMDSSO

cross-linked peptide pairs measured in MS1 were used to determine their relative abundance ratios between unneddylated and neddylated Cul1-Rbx1 complexes for quantitative analysis (Table 1).

Supplementary Table 1: Detailed Summary of DMDSSO Cross-linked Peptides of Cul1-Rbx1 complexes Using LC-MS^n.

Linkage	Linkage Details	MS1 z	MS1 m/z	MS2 m/z	MS2 z	MS2 ppm	MS1 m/z	MS2 m/z	MS2 z	MS2 ppm	Peptide Sequence	Protein	AA #
Cul1 - Cul1	Cul1: 63 - Cul1: 131	4					691.1025	448.738	2	2.3	QSM(Oxidation)AK(Alkene)SR	Cul1	63
								924.464	2	0.9	FSSK(Thiol)VLN(Deamidated)GIC(Carbamidomethyl)AYLNR	Cul1	131
	Cul1: 337 - Cul1: 750	4	493.0421	600.822	2	6.2	495.5573	603.339	2	8.3	IQDGLGELK(Thiol)K	Cul1	337
				376.258	2	1.4		378.775	2	4.9	VPVIK(Alkene)K	Cul1	750
	Cul1: 410 - Cul1: 701	5	759.7628	920.434	2	-6.6					FINNNAVTK(Thiol)MAQSSSK	Cul1	410
				645.975	3	3.2					Q(Gln->pyro-Glu)EQETTHK(Alkene)NIEEDRK	Cul1	701
		5	762.7680	920.441	2	1.1					FINNNAVTK(Thiol)MAQSSSK	Cul1	410
				651.650	3	2.4					QEQETTHK(Alkene)NIEEDRK	Cul1	701
	Cul1: 410 - Cul1: 720	4	651.5775	920.443	2	3.2	654.3420	922.957	2	1.4	FINNNAVTK(Thiol)MAQSSSK	Cul1	410
				373.707	2	2.1		376.222	2	0.2	IMK(Alkene)MR	Cul1	720
	Cul1: 410 - Cul1: 743	4	618.8148	920.442	2	2.1	621.3307	922.959	2	3.6	FINNNAVTK(Thiol)MAQSSSK	Cul1	410
				308.185	2	2.3		310.700	2	0.1	FK(Alkene)PR	Cul1	743
		4	622.8141	928.440	2	2.7	625.5818	930.953	2	-0.2	FINNNAVTK(Thiol)M(Oxidation)AQSSSK	Cul1	410
				308.185	2	2.3		310.700	2	0.1	FK(Alkene)PR	Cul1	743
		4	752.9080	920.440	2	0.0					FINNNAVTK(Thiol)MAQSSSK	Cul1	410
				576.371	2	4.2					FK(Alkene)PRVPVIK	Cul1	743
	Cul1: 410 - Cul1: 750	4	652.8524	376.258	2	1.4	655.3687	378.774	2	2.2	FINNNAVTK(Thiol)MAQSSSK	Cul1	410
				920.441	2	1.1		922.954	2	-1.9	VPVIK(Alkene)K	Cul1	750
	Cul1: 417 - Cul1: 689	4	773.3956	802.896	2	4.8	775.9152	805.415	2	8.9	MAQSSSK(Thiol)SPELLAR	Cul1	417
				734.896	2	1.3		737.414	2	4.4	VNINVPMK(Alkene)TEQK	Cul1	689
	Cul1: 417 - Cul1: 689|693	5	815.6091	802.897	2	6.0					MAQSSSK(ThiolSPELLAR	Cul1	417
				828.078	3	5.2					VNINVPMK(Thiol1)TEQK(Thiol2)QEQETTHK	Cul1	689|693
	Cul1: 417 - Cul1: 693	4	798.8813	810.883	2	-8.2					M(Oxidation)AQSSSK(Thiol)SPELLAR	Cul1	417
				777.873	2	-0.5					TEQK(Alkene)QEQETTHK	Cul1	693
	Cul1: 431 - Cul1: 468	4	546.5285	563.768	2	1.4	549.5465	566.286	2	5.5	YC(Carbamidomethyl)DSLLK(Thiol)K	Cul1	431
				520.288	2	4.1		522.802	2	0.8	FYAK(Alkene)MLAK	Cul1	468
	Cul1: 431 - Cul1: 472	4	458.2395	563.768	2	1.4	460.7551	566.284	2	2.0	YC(Carbamidomethyl)DSLLK(Thiol)K	Cul1	431
				343.705	2	1.4		346.221	2	2.2	MLAK(Alkene)R	Cul1	472
		4	443.9836	519.270	2	-0.8	446.4987	537.772	2	-0.3	YCDSLLK(Alkene)K	Cul1	431
				343.705	2	1.4		346.221	2	2.2	MLAK(Alkene)R	Cul1	472
	Cul1: 464 - Cul1: 693	5	693.9347	947.457	2	-1.3					YIEDKDVFQK(Thiol)FYAK	Cul1	464
				518.917	3	-1.9					TEQK(Alkene)QEQETTHK	Cul1	693
	Cul1: 464 - Cul1: 743	4	632.3239	947.457	2	-1.3	634.8438	949.979	2	5.4	YIEDKDVFQK(Thiol)FYAK	Cul1	464
				308.186	2	5.6		310.702	2	6.5	FK(Alkene)PR	Cul1	743
	Cul1: 468 - Cul1: 693	5	529.6614	536.276	2	7.6					FYAK(Thiol)MLAK	Cul1	468
				518.916	3	-3.9					TEQK(Alkene)QEQETTHK	Cul1	693
		4	661.5743				664.0906	522.803	2	2.7	FYAK(Alkene)MLAK	Cul1	468
				793.859	2	-0.5		796.375	2	-0.1	TEQK(Thiol)QEQETTHK	Cul1	693
	Cul1: 468 - Cul1: 743	4	426.7346	536.275	2	5.7					FYAK(Thiol)MLAK	Cul1	468
				308.185	2	2.3					FK(Alkene)PR	Cul1	743
	Cul1: 472 - Cul1: 689	4	555.7946	343.705	2	1.4					MLAK(Alkene)R	Cul1	472
				758.878	2	-0.7					VNINVPM(Oxidation)K(Thiol)TEQK	Cul1	689
		5	638.1315	736.387	1	3.5					MLAK(Sulfenic)R	Cul1	472
				613.316	4	3.2					VNINVPMK(Alkene)TEQKQEQETTHK	Cul1	689
		4	551.7966	750.883	2	2.5	554.3110	753.394	2	-3.7	MLAK(Alkene)R	Cul1	472
				343.705	2	1.4		346.221	2	2.2	VNINVPMK(Thiol)TEQK	Cul1	689
	Cul1: 472 - Cul1: 693	5	458.8307	359.691	2	1.2					MLAK(Thiol)R	Cul1	472
				518.918	3	0.0					TEQK(Alkene)QEQETTHK	Cul1	693
		4	573.7870	343.704	2	-1.6	575.8003	741.417	1	1.6	MLAK(Alkene)R	Cul1	472
				793.859	2	-0.5		796.377	2	2.4	TEQK(Thiol)QEQETTHK	Cul1	693
		4	577.2838	351.702	2	0.0					M(Oxidation)LAK(Alkene)R	Cul1	472
				793.860	2	0.8					TEQK(Thiol)QEQETTHK	Cul1	693
		4	797.1608	686.405	1	4.7					MLAK(Alkene)R	Cul1	472
				817.422	3	7.0					VNINVPMKTEQK(Alkene)QEQETTHK	Cul1	693
	Cul1: 493 - Cul1: 720	4	574.5350	766.351	2	1.8					LK(Thiol)QAC(Carbamidomethyl)GFEYTSK	Cul1	493
				373.707	2	2.1					IMK(Alkene)MR	Cul1	720
	Cul1: 493 - Cul1: 743	4					544.2850	768.867	2	2.2	LK(Thiol)QAC(Carbamidomethyl)GFEYTSK	Cul1	493
								310.701	2	3.3	FK(Alkene)PR	Cul1	743
	Cul1: 493 - Cul1: 750	4					578.3270	768.869	2	4.8	LK(Thiol)QAC(Carbamidomethyl)GFEYTSK	Cul1	493
								378.774	2	2.2	VPVIK(Alkene)K	Cul1	750
	Cul1: 676 - Cul1: 693	5	560.4869	613.836	2	3.7					LYLGYK(Thiol)NKK	Cul1	676
				518.918	3	0.0					TEQK(Alkene)QEQETTHK	Cul1	693
	Cul1: 693 - Cul1: 743	5	444.6213	308.184	2	-1.0	446.6350	310.700	2	0.1	TEQK(Thiol)QEQETTHK	Cul1	693
				529.575	3	-0.7		531.253	3	1.0	FK(Alkene)PR	Cul1	743
		4	555.5247	793.861	2	2.0	558.0410	796.378	2	3.7	TEQK(Thiol)QEQETTHK	Cul1	693
				308.184	2	-1.0		310.701	2	3.3	FK(Alkene)PR	Cul1	743
	Cul1: 701 - Cul1: 708	4	763.1446	920.397	2	2.9	765.6613	922.911	2	1.1	Q(Gln->pyro-Glu)EQETTHK(Thiol)NIEEDR	Cul1	701
				596.893	2	0.9		599.409	2	1.4	K(Alkene)LLIQAAIVR	Cul1	708
		4	767.4011	928.910	2	2.6	769.9200	931.424	2	0.8	QEQETTHK(Thiol)NIEEDR	Cul1	701
				596.895	2	4.3		599.410	2	3.1	K(Alkene)LLIQAAIVR	Cul1	708
	Cul1: 701 - Cul1: 751	4					785.4117	986.959	2	1.5	Q(Gln->pyro-Glu)EQETTHK(Thiol)NIEEDRK	Cul1	701
								574.346	2	3.8	K(Alkene)CIDILIEK	Cul1	751
		4					799.4044	986.960	2	2.5	Q(Gln->pyro-Glu)EQETTHK(Thiol)NIEEDRK	Cul1	701
								602.856	2	2.4	K(Alkene)C(Carbamidomethyl)IDILIEK	Cul1	751

	Cul1: 708 - Cul1: 751	5	703.3976	785.109	3	1.3	705.4105	786.784	3	-1.4	NIEEDRK(Thiol)LLIQAAIVRIMK	Cul1	708
				571.828	2	-0.2		574.345	2	2.1	K(Alkene)CIDILIEK	Cul1	751
	Cul1: 750 - Cul1: 769	3	919.8239	656.403	2	1.3					FKPRVPVIK(Thiol)K	Cul1	750
				1428.654	1	0.8					VDGEK(Alkene)DTYSYLA	Cul1	769
Rbx1 - Rbx1	Rbx1: 19 - Rbx1: 25	4	385.4660	308.177	2	1.1	387.9807	310.693	2	2.1	SGAGK(Alkene)K	Rbx1	19
				453.748	2	2.4		456.262	2	-1.4	RFEVK(Thiol)K	Rbx1	25
Cul1 - Rbx1	Cul1: 493 - Rbx1: 89	4	580.7935	766.351	2	1.8	583.3085	768.867	2	2.2	LK(Thiol)QAC(Carbamidomethyl)GFEYTSK	Cul1	493
				386.231	2	4.6		388.745	2	0.2	WLK(Alkene)TR	Rbx1	89
	Cul1: 493 - Rbx1: 105	5					795.9872	724.363	2	-7.7	LK(Alkene)QACGFEYTSK	Cul1	493
								837.731	3	3.2	TRQVCPLDNREWEFQK(Thiol)YGH	Rbx1	105
	Cul1: 720 - Rbx1: 89	4	396.4616	386.230	2	2.0	398.9775	388.744	2	-2.4	IMK(Alkene)M(Oxidation)R	Cul1	720
				381.703	2	-1.8		384.218	2	-3.6	WLK(Alkene)TR	Rbx1	89
		4	392.4666	373.707	2	2.1					IMK(Alkene)MR	Cul1	720
				402.216	2	1.8					WLK(Thiol)TR	Rbx1	89
		4	400.4610	386.230	2	2.0	402.9761	388.745	2	0.2	IM(Oxidation)K(Alkene)M(Oxidation)R	Cul1	720
				389.702	2	2.2		408.201	2	-4.6	WLK(Alkene)TR	Rbx1	89
	Cul1: 743 - Rbx1: 89	4	359.7025	308.184	2	-1.0					FK(Alkene)PR	Cul1	743
				402.215	2	-0.7					WLK(Thiol)TR	Rbx1	89
	Cul1: 750 - Rbx1: 89	4	393.7385	376.257	2	-1.2	396.2549	378.773	2	-0.4	VPVIK(Alkene)K	Cul1	750
				386.229	2	-0.6		388.745	2	0.2	WLK(Alkene)TR	Rbx1	89
Rbx1 - Nedd8	Rbx1: 25 - Nedd8: 6	4	435.5029	453.746	2	-2.1					RFEVK(Thiol)K	Rbx1	25
				407.755	2	-0.7					VK(Alkene)TLTGK	Nedd8	6
	Rbx1: 89 - Nedd8: 48	4	579.2941	386.231	2	4.6	581.8102	388.745	2	0.2	WLK(Alkene)TR	Rbx1	89
				763.354	2	-1.2		765.871	2	0.5	LIYSGK(Thiol)QMNDEK	Nedd8	48
Cul1 - Nedd8	Cul1: 315 - Nedd8: 6	5	737.9798	952.115	3	-5.6					HLEIFHTEFQNLLDADK(Thiol)NEDLGR	Cul1	315
				407.758	2	6.7					VK(Alkene)TLTGK	Nedd8	6
	Cul1: 337 - Nedd8: 6	4	508.7902	600.820	2	2.9					IQDGLGELK(Thiol)K	Cul1	337
				407.755	2	-0.7					VK(Alkene)TLTGK	Nedd8	6
	Cul1: 410 - Nedd8: 6	4					671.1172	410.272	2	2.5	FINNNAVTK(Thiol)MAQSSSK	Cul1	410
								922.954	2	-1.9	VK(Alkene)TLTGK	Nedd8	6
		4	672.5969	928.438	2	0.6	675.3737	930.953	2	-0.2	FINNNAVTK(Thiol)M(Oxidation)AQSSSK	Cul1	410
				407.755	2	-0.7		410.272	2	2.5	VK(Alkene)TLTGK	Nedd8	6
	Cul1: 410 - Nedd8: 48	4					841.1733	922.959	2	3.6	FINNNAVTK(Thiol)MAQSSSK	Cul1	410
								749.885	2	0.6	LIYSGK(Alkene)QMNDEK	Nedd8	48
	Cul1: 464 - Nedd8: 6	4	682.1061	407.755	2	-0.7	684.6263	410.272	2	2.5	YIEDKDVFQK(Thiol)FYAK	Cul1	464
				947.458	2	-0.2		949.974	2	0.1	VK(Alkene)TLTGK	Nedd8	6
	Cul1: 468 - Nedd8: 6	4	476.5132	536.269	2	-5.5					FYAK(Thiol)MLAK	Cul1	468
				407.754	2	-3.1					VK(Alkene)TLTGK	Nedd8	6
	Cul1: 493 - Nedd8: 6	4	591.5557	766.351	2	1.8	594.0714	768.866	2	0.9	LK(Thiol)QAC(Carbamidomethyl)GFEYTSK	Cul1	493
				407.756	2	1.8		410.272	2	2.5	VK(Alkene)TLTGK	Nedd8	6
		3	769.3981	721.855	2	3.0					LK(Alkene)QACGFEYTSK	Cul1	493
				846.478	1	3.1					VK(Thiol)TLTGK	Nedd8	6
	Cul1: 493 - Nedd8: 48	4	761.3638	750.367	2	4.6	763.8782	752.880	2	1.0	LK(Alkene)QAC(Carbamidomethyl)GFEYTSK	Cul1	493
				747.368	2	-1.2		749.886	2	1.9	LIYSGK(Alkene)QMNDEK	Nedd8	48
		4	747.1072	721.855	2	3.0	749.6219	724.367	2	-2.1	LK(Alkene)QACGFEYTSK	Cul1	493
				763.355	2	0.1		765.867	2	-4.7	LIYSGK(Thiol)QMNDEK	Nedd8	48
	Cul1: 693 - Nedd8: 6	4	605.3106	793.862	2	3.3	607.8278	796.375	2	-0.1	TEQK(Thiol)QEQETTHK	Cul1	693
				407.757	2	4.2		410.271	2	0.1	VK(Alkene)TLTGK	Nedd8	6
	Cul1: 701 - Nedd8: 6	5	560.6817	656.629	3	-2.0	562.6959	658.310	3	3.9	Q(Gln->pyro-Glu)EQETTHK(Thiol)NIEEDRK	Cul1	701
				407.754	2	-3.1		410.275	2	9.8	VK(Alkene)TLTGK	Nedd8	6
		5	661.3361	824.388	3	1.1					TEQKQEQETTHK(Thiol)NIEEDRK	Cul1	701
				407.756	2	1.8					VK(Alkene)TLTGK	Nedd8	6
		4	700.6001	984.443	2	1.2	703.1206	986.959	2	1.5	Q(Gln->pyro-Glu)EQETTHK(Thiol)NIEEDRK	Cul1	701
				407.755	2	-0.7		410.271	2	0.1	VK(Alkene)TLTGK	Nedd8	6
		5	564.0879	662.307	3	1.8	566.0989	663.982	3	-1.4	QEQETTHK(Thiol)NIEEDRK	Cul1	701
				407.756	2	1.8		410.271	2	0.1	VK(Alkene)TLTGK	Nedd8	6
		6	551.2783	618.543	4	1.4					TEQKQEQETTHK(Thiol)NIEEDRK	Cul1	701
				407.756	2	1.8					VK(Alkene)TLTGK	Nedd8	6
		5	635.7173	781.694	3	6.7					TEQKQEQETTHK(Thiol)NIEEDR	Cul1	701
				407.756	2	1.8					VK(Alkene)TLTGK	Nedd8	6
		4	826.6705	813.735	3	6.5					TEQKQEQETTHK(Alkene)NIEEDRK	Cul1	701
				846.480	1	5.5					VK(Thiol)TLTGK	Nedd8	6

[shaded box] Used in final quantitation
